# A Rare Tumor in the Cervical Sympathetic Trunk: Ganglioneuroblastoma

**DOI:** 10.1155/2016/1454932

**Published:** 2016-11-14

**Authors:** Ozan Erol, Alper Koycu, Erdinc Aydin

**Affiliations:** Department of Otorhinolaryngology, Baskent University Faculty of Medicine, 06500 Ankara, Turkey

## Abstract

Ganglioneuroblastoma is a rare tumor with moderate malignancy, which is composed of mature ganglion cells and seen in sympathetic ganglia and adrenal medulla. The diagnosis is possible after cytological and immunohistochemical studies following a needle biopsy or surgical excision. There is no consensus regarding the need for chemo- or radiotherapy after surgery. In this case report, clinical behavior and diagnosis and treatment of the rare tumor cervical ganglioneuroblastoma were discussed.

## 1. Introduction

Neuroblastomas are malignant solid tumors. They emerge from out-of-control, immature cells of the sympathetic nervous system. Neuroblastomas can emerge in any place where sympathetic nervous tissues are present. They are most commonly seen in suprarenal gland and areas called truncus sympathicus on neural networks in each side of the spine. In cases where truncus sympathicus is involved, neuroblastomas can emerge in either side of the spine and in any area of it [1]. Different levels of microscopic differentiations are observed in the majority of neuroblastomas. The tumor may transform into ganglioneuroma on the basis of fibrous stroma. Various levels of differentiation may be observed between pure neuroblastoma and pure ganglioneuroma and these are called ganglioneuroblastoma [1, 2].

In this study, a 5-year-old patient with no complaints other than neck swelling and a positive pathology of ganglioneuroblastoma is presented.

## 2. Case Report

Five-year-old male patient was admitted to our clinic with complaint of swelling in the left side of the neck, which was not accompanied by any pain for four months. He had no other complaints like loss of weight, fever, night sweating, respiratory distress, or difficulty swallowing. In the physical examination, a hard, mobile mass with an approximate size of 2*∗*3 cm was detected on the front of musculus sternocleidomastoideus in patient's neck. A neck ultrasonography, which had been conducted in the external center, had the result of a lymphadenopathy with the size of 28*∗*21 mm and with no fat hilus on musculus sternocleidomastoideus anterior.

**Figure 1:**
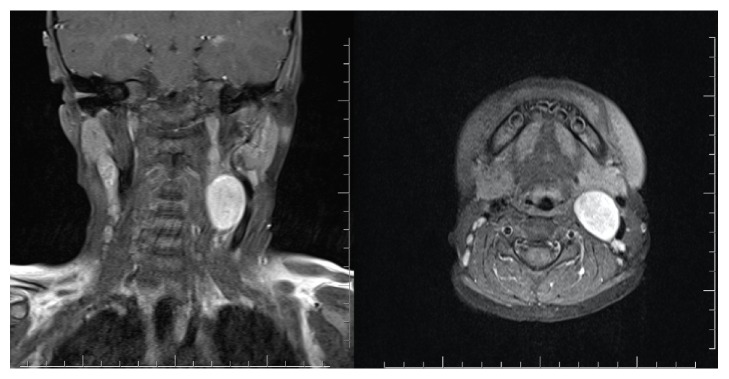
MRI of the head and neck: axial and coronal views of mass. The lesion with 2,8*∗*2,3 cm size on the front of musculus sternocleidomastoideus and internal carotid artery.

Magnetic resonance imaging of the neck showed a properly limited lesion with 2,8*∗*2,3 cm size on the front of musculus sternocleidomastoideus and internal carotid artery. The lesion which pushes parotid gland towards the front was starting from the inferior part of the posterior digastric muscle and progressing up to the C4 level (Figure 1).

A fine-needle aspiration biopsy was offered but refused by the family. In his examination 1 month later, the mass in the neck was found to be approximately 4*∗*4 cm. Patient's family was informed that the mass to be extracted by surgery is deep under large arteries in the neck, that it may cover nerves outside the lymph node, and that there are risks of possible lowering of the left eyelid and in operation of the left vocal cord. The family gave written consent for the surgery. The patient underwent the operation with provisional diagnosis of lymphadenopathy and a tumor of nerve origin.

**Figure 2:**
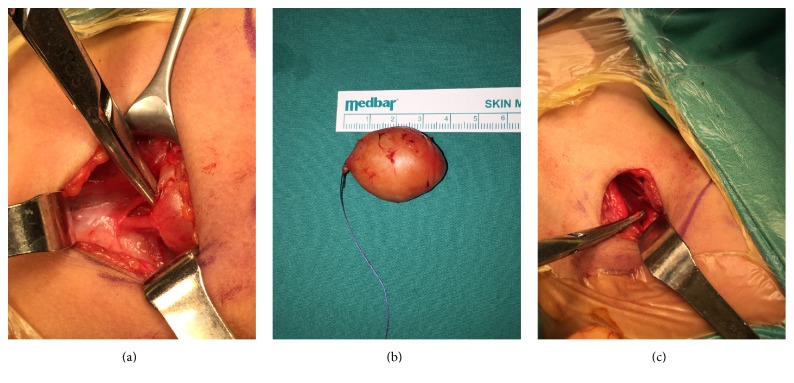
(a) Intraoperative view of the neurogenic mass. (b) The lesion with 4 cm size after the total excision. (c) Upper part of the nerve was held by the clamp.

The properly limited and encapsulated mass on musculus sternocleidomastoideus, which was pushing the carotid artery and internal jugular vein into lateral position, was separated from surrounding tissue by blunt dissection. It was observed that the mass had nerve continuity in its upper and lower regions and it was in fact of nerve origin. Nerves in the upper part were connected by clamping and the nerves which were severed during blunt dissection were made into another specimen. The total mass was extracted (Figure 2).

**Figure 3:**
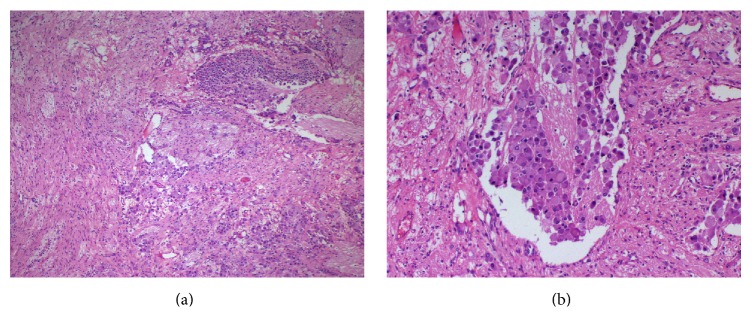
Photomicrographs showing more than 50% of tumor tissue in this case composed of Schwannian stroma and neuropil-like islands, differentiating neuroblasts, and immature and dysplastic ganglion cells. H&E (a and b), original magnification (a) 10x and (b) 40x.

Pathological diagnosis was reported as intermixed type ganglioneuroblastoma. While there was no calcification on the encapsulated tumor which was rich in terms of stroma Schwann cells, it was found that ganglioneuromatosis component is over 50%. In addition, differentiation neuroblasts and immature and dysplastic ganglion cells were observed under microscopic examination (Figure 3).

Patient had developed ipsilateral Horner syndrome (miosis, ptosis, and anhidrosis) in the postoperative period. However, no sign of this was found in the 3rd-month control examination. Tests like abdominal and thorax imaging, complete blood count, bone marrow aspiration test, 24-hour urinary VMA, and N-myc protooncogene protein scan were conducted. No pathological findings were observed. After the I-131 Metaiodobenzylguanidine (I-131 MIBG) scan in the 6th-month control examination, an involvement was observed in the right cervical region, the opposite area of the operated region, and patient was directed to adjuvant chemotherapy. No evidence of any recurrence was found in the 12th-month control examination.

## 3. Discussion

Cervical masses are common pathologies in the pediatric population. While most of them are benign such as inflammatory or congenital ones, over 10% of biopsies extracted from suspicious masses are being reported as malign [2]. Ganglioneuroblastomas represent a histological subgroup of rare neuroblastic tumors with moderate malignancy potential, which originate from neural crest progenitor cells of sympathetic nerve cells. They are very difficult to diagnose using only physical examination and imaging methods. Generally, final diagnosis is possible after cytological and immunohistochemical studies following a surgical excision.

Neuroblastic tumors are divided into four types in International Neuroblastoma Pathology Classification: neuroblastoma, intermixed ganglioneuroblastoma, modular ganglioneuroblastoma, and ganglioneuroma [3]. The type our case study reports, intermixed ganglioneuroblastoma, was reported in several studies in the literature. While in those publications this type of mass was reported to cause respiratory distress and difficulties in swallowing, our case study reported none of these symptoms.

The International Neuroblastoma Risk Group (INRG) classification system was developed to establish a consensus approach for pretreatment risk stratification. The International Neuroblastoma Staging System (INSS) is a postsurgical staging system. In the INRG classification system, a combination of clinical, pathologic, and genetic markers is used to predict whether the tumor will grow and how it will respond to treatment. These markers are used to define risk. Using the following factors, neuroblastoma is classified into 1 of 4 categories: very low-risk, low-risk, intermediate-risk, or high-risk. INRG includes the following: the stage of the disease according to the INRG Staging System, age at the time of diagnosis, histologic category, such as maturing ganglioneuroma versus ganglioneuroblastoma, intermixed versus ganglioneuroblastoma, or nodular versus neuroblastoma, grade or how cells of the tumor are differentiated, MYCN gene status, chromosome 11q status, and tumor cell ploidy, which is the DNA content of tumor cells.

The INRG Staging System (INRGSS) was designed specifically for the INRG pretreatment classification system. It does not include surgical results or spread to lymph nodes to determine the stage. Knowledge regarding the presence or absence of image defined risk factors (IDRFs) is required for this staging system. Stage L1: the tumor is located only in the area where it started; no IDRFs are found on imaging scans, such as CT or MRI. Stage L2: the tumor has not spread beyond the area where it started and the nearby tissue; IDRFs are found on imaging scans, such as CT or MRI. Stage M: the tumor has spread to other parts of the body. Stage MS: the tumor has spread to only the skin, liver, and/or bone marrow (less than 10% bone marrow involvement) in patients younger than 18 months [4].

Al-Jassim had stated that in his case study of 2-year-old patient with ganglioneuroblastoma the patient had developed postoperative Horner syndrome, but this had been resolved spontaneously over time [5]. Moukheiber et al. had reported in their study that 3 patients had primary cervical ganglioneuroblastoma and developed nerve paresis in the postoperative period [6]. Due to the risk of postoperative nerve palsy being high, parents and children should be informed in detail before the operation. As it happened in our case study, if the mass was found to be of nerve origin, the parents should absolutely be informed about possible complications (Horner syndrome due to sympathetic nerve chain incision, respiratory and nutrition problems due to vagal nerve incision, etc.) and their consent should be taken.

There is no doubt that imaging techniques are not enough for a definitive diagnosis. Magnetic resonance imaging is recommended. Ganglioneuroblastoma is typically heterogenous in imaging. Generally it shows high signal intensity in T1 images and low signal intensity in T2 images [5, 6]. However, as it happened in our case, it may show hypointense characteristics in T1 and hyperintense characteristics in T2, which may lead to misdiagnosis. First, a fine-needle biopsy should be offered for diagnosis. Following that, a staged treatment plan may be tailored according to histopathological diagnosis and body scan results [6, 7]. In our case, our patient's parents initially refused our suggestion of fine-needle aspiration and stated that they want to continue monitoring the situation. However, after the mass was found to have grown in size during control examination, patient was offered excisional biopsy with a prediagnosis of lymphadenopathy. After finding that the mass is of nerve origin, it was totally extracted and the examination of metastasis or primary tumor was conducted in postoperative period.

It is known that most neurogenic tumors secrete catecholamine. High levels of catecholamine may be an indication of recurrence or the presence of primary tumor. Genetic characteristics are also associated with tumor behavior. N-myc is a protooncogen, where its chromosome arm resides in far end of 2p. Detection of multiple N-myc copies indicates a fast tumor growth and negative prognosis in patients who show these histological characteristics and are over 1 year of age. Also a scintigraphic scan with MIBG should be conducted and invasion should be determined. In our patient, vanilmandelic acid (VMA) and N-myc protooncogene were found to be negative during the postoperative period [6–8].

The need for chemotherapy in postoperative period can be decided after a systematic scan was conducted. In one literature study, a myelogram of a patient, who was under 1 year of age, had showed metastatic cells and the patient had underwent postoperative chemotherapy. Some authors advocate giving chemotherapy even when there is no metastasis, due to ganglioneuroblastoma being a malignant tumor in nature [6–8]. In our patient, while there was no evidence of recurrence until the 6th month after the operation, PET-BT scan at the 6th month showed an involvement in the right side of the neck, and patient was given chemotherapy. After 2 sessions of chemotherapy, no recurrence was found in the 12th-month control examination of the patient.

## 4. Conclusion

In this case study, our aim was to discuss clinical behavior and diagnosis and treatment of primary cervical ganglioneuroblastoma together with the literature review. Ganglioneuroblastomas in cervical ganglions are rather rare tumors. They are usually asymptomatic, thus making preoperative diagnosis difficult. Clinicians should bear this in mind as this is found in the differential diagnosis of masses in parapharyngeal space.
